# Prevalence of antibiotic prescription in pediatric outpatients in Italy: the role of local health districts and primary care physicians in determining variation. A multilevel design for healthcare decision support

**DOI:** 10.1186/s12889-017-4905-4

**Published:** 2017-11-17

**Authors:** Mirko Di Martino, Adele Lallo, Ursula Kirchmayer, Marina Davoli, Danilo Fusco

**Affiliations:** Department of Epidemiology, Lazio Regional Health Service, Via Cristoforo Colombo, 112 - 00147 Roma, Italy

**Keywords:** Antibiotic prescription patterns, Pediatric outpatients, Preschool children, Geographic variation, Multilevel models

## Abstract

**Background:**

According to scientific literature, antibacterials are prescribed for common pediatric conditions that do not benefit from antibiotic therapy. The link between antibiotic use and bacterial resistance is well known. Antibiotic overprescribing generates high social costs and severe consequences for children. Our objectives were to analyze antibiotic prescription patterns in pediatric outpatients residing in the Lazio region (Italy), to identify physicians’ characteristics associated with increased propensity for prescription, to identify the priority axes for action to improve the rational use of antibiotics.

**Methods:**

We enrolled all children aged 13 years or less in 2014. Antibiotic prescription patterns were analyzed during a one-year follow-up period. The main outcome measures were the antibiotic prescription prevalence, and the geographic variation in antibiotic prescribing. Multilevel models were performed to analyze variation. Variation was expressed as Median Odds Ratios (MORs). If the MOR is 1.00, there is no variation between clusters. If there is considerable between-cluster variation, the MOR will be large.

**Results:**

We enrolled 636,911 children. Most of them were aged 6–13 years (57.3%). In 2015, the antibiotic prescription prevalence was 46% in the 0–13, 58% in the 0–5, and 37% in the 6–13 age group. Overall, penicillins were the most prescribed antibiotics, their consumption increased from 43% to 52% during the 2007–2015 period. In 2015, the antibiotic prescription prevalence ranged from 30% to 62% across local health districts (LHDs) of the region. Moreover, a significant (*p* < 0.001) variation was observed between physicians working in the same LHD: MORs were equal to 1.52 (1.48–1.56) and 1.46 (1.44–1.48) in the 0–5 and 6–13 age groups, respectively. The probability of prescribing antibiotics was significantly (p < 0.001) lower for more-experienced physicians.

**Conclusions:**

Pediatric antibiotic use in the Lazio region is much higher than in other European countries. The intra-regional drug prescribing variability underlines the lack of therapeutic protocols shared at regional level and raises equity issues in access to optimal care. Both LHD managers and individual physicians should be involved in training interventions to improve the targeted use of antibiotics and mitigate the effect of contextual variables, such as the spatial-related socioeconomic status of the patient/parent binomial.

## Background

Antibiotics are the most widely prescribed therapeutic agents in children. The prevalence of antibiotic prescriptions differs across age, with preschool children being more likely to be prescribed antibiotics [1]. It is well recognized that antibacterials are prescribed to children for the treatment of common pediatric conditions, such as bronchitis, upper-respiratory-tract infections, colds, that do not benefit from antibiotic therapy [2, 3]. It is even more worrying that approximately 50% of antibiotic prescriptions for children given by primary care physicians are not necessary [4]. The link between antibiotic use and bacterial resistance is well known [5] thus antibiotic overprescribing has a significant impact on public health. In fact, antibiotic resistance is a growing international threat, with high social costs for communities and severe consequences, such as failure to respond to treatment, prolonged illness, and greater risk of complications and mortality [2, 6]. In Italy, in order to stem this problem, the antibiotic resistance surveillance project “AR-ISS” was launched in 2001 [7]. However, there is not yet a national plan against antibiotic resistance. The pediatric antibiotic prescription prevalence in Italy was almost fourfold higher than in the United Kingdom (52% vs 14%, respectively), and the prescription rate was fourfold higher than in Denmark and The Netherlands (1.3 vs 0.3 prescriptions/person/year) [2]. Moreover, within-country differences were detected among Italian geographic areas and primary care physicians [2]. This heterogeneity raises equity concerns in access to optimal care. In Italy, a central role in the governance of primary-care prescription patterns is played by the local health district (LHD), a body delegated by the National Health System to provide health care to a specific geographic area. Each LHD is composed of a well-defined group of primary care physicians (including general practitioners and pediatricians), sharing the same clinical guidelines and participating in the same learning interventions, coordinated by a district director. Acting in this framework, LHDs and physicians may have a synergistic effect on variation in antibiotic prescription. In fact, physicians are influenced by the groups to which they belong, and the properties of those groups are in turn influenced by the physicians who make up that group. From current scientific evidence it is not possible to measure how much of the variation in prescription patterns is attributable to the LHD characteristics and how much to the different prescribing habits between physicians. However, from a public health perspective, it is crucial to understand whether antibiotic prescribing choices are driven by LHD features, e.g. the organizational structure and the geographic location, or by characteristics related to primary care physicians working within the same LHD, such as gender, years of professional experience, and organizational arrangement. Therefore, the analysis of these ‘components of variation’ may be a useful tool for policy makers, in order to identify the more effective interventions to enhance the rational use of antibiotics and equity in health care delivery.

### Objectives

The objectives of this study are as follows: 1) to measure the antibiotic prescription prevalence in pediatric outpatients of the Lazio region, Italy; 2) to describe the distribution of prescriptions by antibiotic class; 3) to identify physicians’ characteristics associated with increased propensity for antibiotic drug prescriptions; 4) to identify the priority axes for action aimed at improving the rational use of antibiotic drugs.

## Methods

### Data sources

This is a retrospective, observational, population-based study, conducted on the population living in the Lazio region of Italy, which comprises approximately 5 million persons. Patients were recruited from individuals registered with the LHDs of the region, who were eligible over the study period. The information on patients and their assigned physicians was retrieved from the regional Health Assistance Register. Drug information was collected from the regional Drug Claims Register, which was described in detail elsewhere [8]. Data available on each prescription includes the patient’s identification number, the prescribing physician’s number, the Anatomical-Therapeutic-Chemical (ATC) code of the drug purchased, the number of packs, the number of units per pack, the dosage, the unit cost per pack and the prescription date. The drugs under study are equally available for all residents, in accordance with the universal health-care insurance coverage. All prescriptions are recorded electronically in the Drug Claims Register. Using a unique and anonymous subject identifier, drug information was cross-linked to patient and physicians characteristics. Any date of death was retrieved from the Mortality Information System.

### Study population and drug exposure

The study included all children aged 13 years or less on December 31, 2014 (the *index date*). Antibiotic (ATC code J01) prescription patterns were analyzed during a one-year follow-up period, starting from the index date. Therefore, the main analysis was performed using data from 2015, however, the same enrollment criteria were applied to the period 2007–2014, to describe and compare the “cross-sectional” antibiotic prescription patterns over time. Drug exposure was measured by the prescription prevalence, expressed as the number of people who received at least one prescription during follow-up, per 100 individuals in the population [2]. The distribution of prescriptions by antibiotic class was described, considering penicillins, cephalosporins and macrolides. We assessed the temporal trend in prescription patterns by analyzing the prescription prevalence and the distribution of prescriptions by antibiotic class for the years from 2007 to 2015. It is worth noting that in some countries children can be cared for by family pediatricians or general practitioners. In Italy, children are assigned to a pediatrician until they are 6 years old; afterwards, parents can choose to remain with that pediatrician until the child is 14 years old or to register the child with a general practitioner. All adolescents over 14 years of age are assigned to a general practitioner [2]. Antibiotic prescribing habits of pediatricians and general practitioners were compared in terms of antibiotic prescription prevalence and antibiotic classes prescribed. Detailed analyses were also performed on preschool children, defined as children aged less than or equal to 5 years, and on children aged 6–13 years.

### Statistical methods and analysis of variation

Continuous variables were presented as mean value ± standard deviation A global chi-square test was performed in order to check for differences in antibiotic prescription prevalence during the 2007–2015 period. A map of the Lazio region was produced to show and compare the antibiotic prescription prevalence by LHD in 2015. The classes used in the map were calculated applying the Jenks natural breaks optimization algorithm [9], which reduces the variance within classes and maximizes the variance between classes. Linear regression analysis was performed in order to measure the association between second-choice antibiotic prescription (e.g., cephalosporins) and prevalence of antibiotic prescription in the primary care physician population. In order to avoid difficulties when applying linear regression models to bounded-range dependent variables, the logit transformation was applied to the response variable before estimating the model [10]. “Intercept-only” logistic multilevel models [11] were performed in order to analyze geographic variation, by measuring and comparing the variability in antibiotic prescription patterns attributable to LHDs and primary care physicians. Multilevel models analyze the healthcare system as if it were a “hydraulic system”. If there are some “leaks”, this approach allows us to locate where they are and prioritize interventions. The variance components were expressed in terms of Median Odds Ratios (MORs). The MOR quantifies the variation between clusters by comparing two persons from two randomly chosen, different clusters. Consider two persons with the same covariates, chosen randomly from two different clusters. The MOR is the median odds ratio between the person of higher propensity and the person of lower propensity. This measure is always greater than or equal to 1.00. If the MOR is 1.00, there is no variation between clusters. If there is considerable between-cluster variation, the MOR will be large [12]. Multilevel models were also applied to identify physicians’ characteristics associated with increased propensity for antibiotic drug prescriptions. The full list of evaluated characteristics was as follows: age, gender, years of professional experience, and organizational arrangement (none, association, network, group practice) [13]. Multilevel models were separately performed in the 0–5 and 6–13 age groups. Odds ratios (ORs), 95% confidence intervals (95% CIs) and *p*-values were reported. The Akaike Information Criterion (AIC) was used to determine the model that provided the best account of the data. In fact, AIC deals with the trade-off between the goodness-of-fit and the complexity of the model. The “best” model is the one with minimum AIC value [14]. All analyses were performed using SAS statistical software (version 9.2).

## Results

We analyzed 636,911 children, 51.4% of them were male. A total of 271,780 children were aged 0–5 years (42.7%), whereas 365,131 were aged 6–13 years (57.3%).

**Fig. 1:**
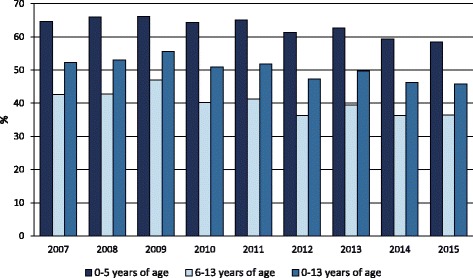
Pediatric antibiotic prescription prevalence, by calendar time and age group (0–5, 6–13, and 0–13 years of age). Lazio Region, years 2007–2015

**Fig. 2:**
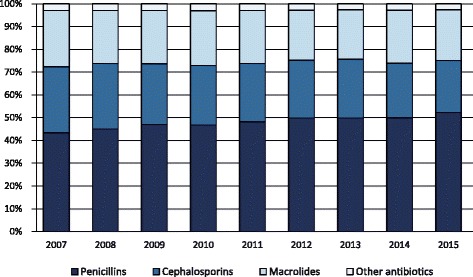
Percentage distribution (percentage of total prescriptions) of the most prescribed antibiotic classes in the 0–13 age group, by calendar time. Lazio Region, years 2007–2015

**Fig. 3:**
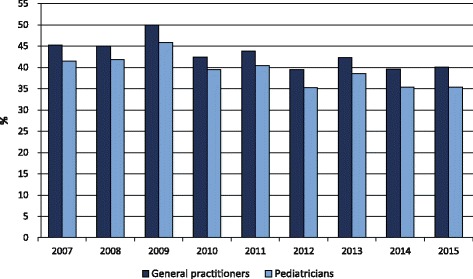
Antibiotic prescription prevalence in the 6–13 age group: a comparison between General practitioners and Pediatricians. Lazio Region, years 2007–2015

**Fig. 4:**
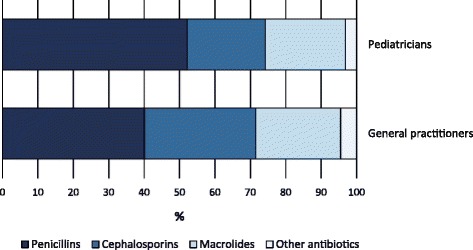
Percentage distribution (percentage of total prescriptions) of the most prescribed antibiotic classes in the 6–13 age group: a comparison between General practitioners and Pediatricians. Lazio Region, 2015

**Fig. 5:**
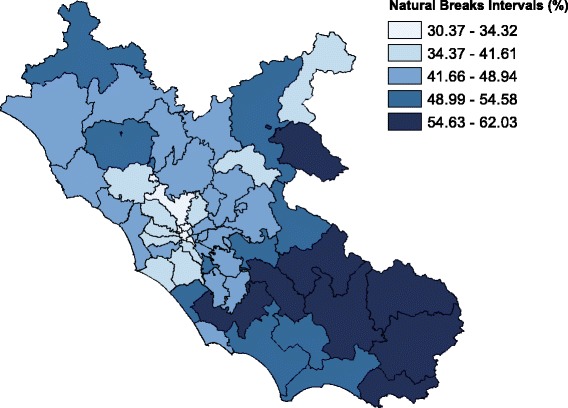
Variation in antibiotic prescription prevalence in the 0–13 age group, by local health district. Lazio Region, 2015

In 2015, the antibiotic prescription prevalence in the Lazio region was about 46% in the 0–13 age group, 58% in the 0–5, and 37% in the 6–13 age group. Figure 1 shows the prescription prevalence by age group for the period 2007–2015. The time-trend analysis indicated a slight but statistically significant decrease in the last 3 years for the 0–13 (*p*-value <0.001) and 0–5 (p-value <0.001) age groups. The highest values were observed in 2009: almost 56% in the 0–13 age group, more than 66% in preschool children, and 47% in the 6–13 age group. Figure 2 shows the percentage distribution of the most prescribed antibiotic classes from 2007 to 2015, in the 0–13 age group. Penicillins were the most prescribed antibiotics and their consumption, expressed as the percentage of total antibiotic prescriptions, increased from 43% to 52% over time. During the same period, cephalosporins decreased from 29% to 23%, whereas macrolides decreased from 25% to 22%. The percentage distribution in preschool children was very similar to that observed in the 0–13 age group, showing a slightly higher percentage in penicillin prescriptions, 54% versus 52%, in 2015. Children were cared by 5097 primary care physicians. About 63% of physicians were males, and the mean age was 58 ± 6 years. Among primary care physicians, 4323 were general practitioners and 774 were pediatricians. The distribution of physicians according to the organizational arrangement was as follows: none = 20%, association = 9%, network = 36%, and group practice = 35%. It is worth noting that, in both 0–5 and 6–13 age groups, second-choice antibiotic drugs (e.g., cephalosporins) were more commonly prescribed by physicians characterized by a high prevalence of antibiotic prescription. We found that this correlation was positive and statistically significant (*p*-value <0.001). In the 0–5 age group, for each 10-percentage-point increment in antibiotic prescription prevalence, the propensity of prescribing cephalosporins increased by 2 percentage points. In the 6–13 age group, for each 10-percentage-point increment in antibiotic prescription prevalence, the propensity of prescribing cephalosporins increased by 2.5 percentage points. The 6–13 age group is particularly interesting. In fact, within this age range, parents can choose to remain with the pediatrician or switch to a general practitioner. This allows comparing differences in antibiotic prescribing habits between pediatricians and general practitioners. In the study period, the antibiotic prescription prevalence in the 6–13 age group was systematically higher for general practitioners (Fig. 3). In 2015, the prescription prevalence was found to be 40% for general practitioners and 35% for pediatricians. The comparison between general practitioners and pediatricians also underlined differences in the percentage distribution of antibiotic classes (Fig. 4). In 2015, penicillins accounted for 40% and 52% of total prescriptions among general practitioners and pediatricians, respectively. A further difference was observed in the percentage of cephalosporins: 32% among general practitioners versus 22% among pediatricians. Moreover, as shown in Fig. 5, a high geographic variation was observed among the 55 LHDs of the Lazio region in the 0–13 age group. In 2015, the antibiotic prescription prevalence ranged from 30% to 62%. In the 0–5 age group the geographical gradient (i.e. the apparently increasing prevalence as one moves away from Rome) was identical however, values were higher. In fact, in preschool children the antibiotic prescription prevalence ranged from 40% to 77% across the LHDs of the region. Results of multilevel models are presented in Table 1. The amounts of variation in prescription prevalence attributable to the LHDs and to the primary care physicians were measured and compared. A relevant variation between LHDs was detected in both 0–5 and 6–13 age groups, MOR = 1.39 (95%CI: 1.30–1.52) and MOR = 1.34 (95%CI: 1.27–1.43), respectively. Moreover, a significant variation was observed between primary care physicians working in the same LHD, MOR = 1.52 (95%CI: 1.48–1.56) in the 0–5 age group and MOR = 1.46 (95%CI: 1.44–1.48) in the 6–13 age group. After the inclusion of variables reflecting physicians’ characteristics (i.e. gender, years of professional experience, and organizational arrangement), model fit was slightly improved. In the 0–5 age group, the AIC moved from 343,161 to 343,144 whereas in the 6–13 age group the AIC moved from 453,904 to 453,897. In both age groups, the probability of prescribing antibiotics was lower for more-experienced physicians. The odds ratio for a 5-year increment in clinical practice experience was 0.92 (95%CI: 0.89–0.95) in the 0–5 age group and 0.97 (95%CI: 0.96–0.99) in the 6–13 age group. Physician’s age was dropped due to collinearity, whereas the other variables (physician’s gender and organizational arrangement) were not statistically significant.

**Table 1:** The role of primary care physicians and local health districts in determining variation. Lazio Region, 2015

Levels of the health care system	Median Odds Ratios^a^	
	0-5 years of age	6-13 years of age
Primary care physician	1.52 (95% CI: 1.48-1.56) *p < 0.001*	1.46 (1.44-1.48) *p < 0.001*
Local health district	1.39 (1.30-1.52) *p < 0.001*	1.34 (1.27-1.43) *p < 0.001*
AIC	343,161	453,904

^a^Intercept-only, logistic multilevel models

## Discussion

In this study of about 637,000 children, we found that, in 2015, the antibiotic prescription prevalence in the Lazio region was about 46% in the 0–13 age group, 58% in preschool children, and 37% in the 6–13 age group. Pediatric antibiotic use in Lazio was in line with other Italian regions, and was much higher than in other European countries [2, 15, 16], despite national and international recommendations to reduce antibiotic use and prevent resistance. However, the time-trend analysis suggested a slight improvement, probably due to the information campaign launched by the Italian Ministry of Health in 2010, aimed at increasing awareness on antibiotic resistance and encouraging best practices in the general population, and among health workers and policy makers. Anyway, this improvement cannot be considered satisfactory. Moreover, the use of second-choice antibiotics is common. Cephalosporins, just to discuss an example, are largely prescribed in Italy, whereas they are very rarely used in other countries such as Denmark and the Netherlands [2, 17]. With regard to physicians’ prescribing habits, as shown in a previous study [2], we found a worrying and significant picture. In fact, second-choice antibiotic prescription and high propensity to prescribe antibiotics were associated to each other. As regards the statistical analysis, results of multilevel models can be very interesting from a policy perspective. First, a relevant geographic variation in the antibiotic prescription prevalence was observed between the LHDs of the Lazio region. Spatial heterogeneity raises equity concerns in access to optimal care and underlines the lack of evidence-based therapeutic protocols shared at regional level. However, we must take into account that geographic variation may also reflect socio-cultural and economic differences related to patients/parents, such as patient’s general condition or socioeconomic status [18]. Second, a high variability was detected among primary care physicians working within the same LHD. This finding support the hypothesis that physicians’ characteristics, both observed and unobserved, may play a major role in determining differences in antibiotic prescribing. In this regard, we found differences between general practitioners and pediatricians in both the prescription prevalence and the percentage distribution of antibiotic classes. Furthermore, the antibiotic prescription prevalence was associated with the physician’s experience. In fact, more experienced physicians showed a lower probability of prescribing antibiotics, suggesting a diagnostic uncertainty problem, especially for the youngest primary care doctors. As regards the priority axes for action to improve the rational use of antibiotics, the median odds ratios related to LHDs and primary care physicians were very similar, very high, and with narrow confidence intervals. Therefore, the two axes are equally important under a policy perspective. Both LHD managers and individual primary care physicians should be involved in educational, training or “audit and feedback” interventions, in order to reduce the geographic variation in antibiotic prescription patterns and decrease inappropriate antibiotic use [19]. Furthermore, given that a relevant portion of variation is attributable to the patient/parent binomial [18, 20], healthcare providers should perform informative and educational campaigns to parents, so that they may contribute to a more rational drug use, also reducing the unjustified call for prescriptions [16].

There are some study limitations to be considered. First, our health information systems do not collect data on indications for which antibacterials are prescribed. Second, the results come from a single region in Italy and may be not generalizable to other geographic areas. However, our findings are in line with results of other studies carried out in different Italian regions [2, 15, 16]. Third, we did not directly analyze the role of parents in antibiotic prescribing decisions, because our information systems do not allow the linkage between the child and his parents.

## Conclusions

Despite international and national guidelines aimed at reducing antibiotic prescribing in pediatric outpatients, antibiotic use in the Lazio region of Italy is still much higher than in other European countries. Moreover, the use of second-choice antibiotics is common. The intra-regional drug prescribing variability underlines the lack of therapeutic protocols shared at regional level and raises equity issues in access to optimal care. Both LHD managers and individual primary care physicians should be involved in training interventions, aimed to improve the targeted use of antibiotics and mitigate the effect of contextual variables, such as the spatial-related socioeconomic status of the patient/parent binomial.

## Abbreviations

AIC: Akaike Information Criterion
ATC: Anatomical Therapeutic Chemical
CI: Confidence Interval
LHD: Local Health District
MOR: Median Odds Ratio
OR: Odds Ratio
